# Clinical dosimetry and efficacy of LED photobiomodulation for chronic lower-limb wound healing: a systematic review of randomized trials

**DOI:** 10.1007/s10103-026-04928-y

**Published:** 2026-06-19

**Authors:** Mariana Bezerra Miranda, Ana Carolina Silva Barros, Ana Vitoria Lima de Paula, Rayana Fontenele Alves, Rebeca Barbosa da Rocha, Vinicius Saura Cardoso

**Affiliations:** 1 Postgraduate Program in Biomedical Sciences, Federal University of the Parnaíba Delta (UFDPar), Parnaíba, Piauí Brazil; 2 Center of Medical Specialties, Parnaíba, Piauí Brazil

**Keywords:** Photobiomodulation, LED, Chronic wounds, Lower limbs, Diabetic foot ulcer, Wound healing

## Abstract

Chronic lower-limb wounds represent a major clinical and socioeconomic burden due to delayed healing and high recurrence rates. Light-emitting diode (LED) photobiomodulation has been proposed as a noninvasive and low-cost adjunctive therapy; however, clinical evidence remains inconsistent. This systematic review aimed to synthesize evidence from randomized clinical trials investigating the efficacy of LED photobiomodulation for chronic lower-limb wound healing and to identify irradiation parameters associated with improved outcomes. A comprehensive search was conducted in PubMed, Scopus, Web of Science, and Embase up to May 2026. Six randomized clinical trials met the inclusion criteria. Wavelengths ranged from 620 to 950 nm, and energy densities varied between 2.4 and 126 J/cm². The findings suggest that LED photobiomodulation may promote wound area reduction, improve wound bed quality, and increase microcirculation, particularly in diabetic foot ulcers. However, one study using a high energy density (126 J/cm²) did not demonstrate beneficial effects, suggesting a possible dose-dependent response. The overall certainty of the evidence, assessed using the GRADE approach, was classified as very low due to inconsistency and indirectness among the included studies. Although LED photobiomodulation appears to be safe and demonstrates therapeutic potential, substantial heterogeneity in irradiation parameters, small sample sizes, and methodological limitations preclude definitive conclusions regarding its clinical efficacy. Well-designed randomized controlled trials with standardized protocols and dose–response investigations are needed to establish optimal therapeutic parameters and confirm clinical efficacy.

## Introduction

Wound healing is a complex and dynamic biological process characterized by coordinated and interdependent phases: inflammation, proliferation, and extracellular matrix remodeling [1]. Disruptions in these processes—whether due to metabolic dysfunction, vascular impairment, or mechanical stress—may delay or impair tissue repair, resulting in chronic non-healing wounds [2]. The loss of skin barrier integrity increases wound susceptibility to bacterial colonization, contributing to increased morbidity, mortality, and healthcare costs [3, 4].

Wound management represents a substantial economic burden, with estimated healthcare expenditures reaching US$96.8 billion, including approximately US$38.3 billion associated with wounds complicated by infection [5]. Despite the magnitude of this problem and its significant impact on morbidity and mortality, effective therapeutic strategies remain limited [6]. Conventional treatment approaches are associated with high costs and suboptimal outcomes, with only 35% of wounds achieving complete healing within a mean period of 4.4 months [7].

Among current therapeutic approaches, photobiomodulation (PBM) using light-emitting diodes (LED) has emerged as a low-cost and highly portable modality capable of treating large tissue areas [8]. An LED are semiconductor devices that emit polychromatic, noncoherent, and typically non-collimated light, allowing greater beam dispersion and more uniform tissue irradiation [9]. Photon absorption by cytochrome c oxidase (CCO) triggers cellular signaling cascades involved in cell proliferation, angiogenesis, and extracellular matrix synthesis. These effects promote cell migration, collagen deposition, and progression through the proliferative and remodeling phases of wound healing [10–13].

However, the effects of LED photobiomodulation are highly dependent on parameters such as wavelength, energy density, power output, exposure time, application frequency, and the distance between the device and the tissue [14]. Although in vitro studies and animal models provide relevant mechanistic evidence regarding the effects of LEDs on wound healing [15–17], the extrapolation of these findings to human skin remains limited. Variations in tissue thickness, extracellular matrix composition, vascularization, and immune response influence light absorption and consequently affect the biological efficacy of PBM [18].

In cell cultures, light irradiation is applied directly to isolated cells in the absence of tissue barriers, whereas animal models typically exhibit thinner skin, higher metabolic rates, and faster inflammatory responses [19, 20]. In contrast, human skin—particularly in chronic wounds—is characterized by hyperkeratosis, ischemia, bacterial biofilm, and microangiopathy, factors that may reduce light penetration and compromise the therapeutic efficacy of PBM [21].

In this context, the objective of this systematic review is to analyze the efficacy of photobiomodulation with LEDs in the healing of chronic lower-extremity wounds and to identify the most effective irradiation parameters. To the best of our knowledge, this is the first systematic review to specifically investigate the effects of LED photobiomodulation on the repair of chronic lower-extremity wounds based on clinical trials.

## Methods

The methods of this systematic review were predefined in accordance with the recommendations of the Preferred Reporting Items for Systematic Reviews and Meta-Analyses (PRISMA) guidelines [22]. The study protocol was registered with the International Prospective Register of Systematic Reviews (PROSPERO) under the registration number CRD420251162401.

### Eligibility criteria

The guiding research question was: “In patients with lower limb wounds, does LED photobiomodulation improve healing compared to conventional treatment, placebo, or other phototherapy modalities?”. Eligibility criteria were structured according to the PICOS strategy (Table 1). Studies investigating the effects of LED on the healing of lower-extremity wounds were included, with no restrictions regarding publication period or language. Studies that did not specify irradiation parameters, combined LED with other physical therapies, invasive and/or pharmacological interventions, or did not focus primarily on cutaneous wound healing were excluded. Studies without a comparator group were also excluded, as well as systematic reviews, narrative reviews, case reports, letters to the editor, and observational or experimental studies.

**Table 1:** Definition of the PICOS question

P	Patients with lower limb wounds People aged 18 or older
I	Photobiomodulation with LED
C	Standard care, placebo, or other phototherapy and treatment modalities
O	*Main results*: Parameters related to wound healing, including wound area reduction, healing rates, inflammation, and pain
S	Randomized clinical trials

### Information sources and search strategy

A comprehensive search was conducted in the following databases: PubMed, Scopus, Web of Science, and Embase. The most recent search was performed in May 2026. The search strategy was developed based on the previously defined PICOS framework, incorporating controlled and uncontrolled terms combined using Boolean operators. The search terms included: wound healing, wounds and injuries, ulcer, skin ulcer, skin lesions, tissue repair, excisional wound, pressure ulcer, diabetic ulcer, diabetic foot, skin burn, burn wound, acute wound, chronic wound, open wound, cutaneous wound, light emitting diode, light-emitting diode, non-coherent light, NASA light-emitting diode, and LED therapy. Additionally, a manual search was performed by screening the reference lists of the included studies and by using the search terms independently. Detailed search strategies for each database are presented in Table 2.

**Table 2:** Search strategy used in the databases included in the study

Databases	Search strategy
PUBMED	#1 (((((((((“Wound Healing“[Title/Abstract]) OR (“Wounds and Injuries“[Title/Abstract])) OR (“Ulcer*“[Title/Abstract])) OR (“Skin Ulcer“[Title/Abstract])) OR (“Skin lesions“[Title/Abstract])) OR (“Tissue repair“[Title/Abstract])) OR (“pressure ulcer“[Title/Abstract])) OR (“diabetic ulcer“[Title/Abstract])) OR (“Skin burn“[Title/Abstract])) OR (“Wound*“[Title/Abstract]) AND #2 ((((“Light Emitting Diode*“[Title/Abstract]) OR (“light-emitting diode“[Title/Abstract])) OR (“Non-coherent light“[Title/Abstract])) OR (“NASA light-emitting diode“[Title/Abstract])) OR (“LED therapy“[Title/Abstract])
SCOPUS	#1 (TITLE-ABS-KEY (“Wound Healing”) OR TITLE-ABS-KEY (“Wounds and Injuries”) OR TITLE-ABS-KEY (“Ulcer*”) OR TITLE-ABS-KEY (“Skin Ulcer”) OR TITLE-ABS-KEY (“Skin lesions”) OR TITLE-ABS-KEY (“Tissue repair”) OR TITLE-ABS-KEY (“pressure ulcer”) OR TITLE-ABS-KEY (“diabetic ulcer”) OR TITLE-ABS-KEY (“Skin burn”) OR TITLE-ABS-KEY (“Wound*”)) AND #2 (TITLE-ABS-KEY (“Light Emitting Diode*”) OR TITLE-ABS-KEY (“light-emitting diode”) OR TITLE-ABS-KEY (“Non-coherent light”) OR TITLE-ABS-KEY (“NASA light-emitting diode”) OR TITLE-ABS-KEY (“LED therapy”))
WEB OF SCIENCE	#1 (((((((((TS=(“Wound Healing”)) OR TS=(“Wounds and Injuries”)) OR TS=(“Ulcer*”)) OR TS=(“Skin Ulcer”)) OR TS=(“Skin lesions”)) OR TS=(“Tissue repair”)) OR TS=(“pressure ulcer”)) OR TS=(“diabetic ulcer”)) OR TS=(“Skin burn”)) OR TS=(“Wound*”) AND #2 ((((TS=(“Light Emitting Diode*”)) OR TS=(“light-emitting diode”)) OR TS=(“Non-coherent light”)) OR TS=(“NASA light-emitting diode”)) OR TS=(“LED therapy”) #1 ‘wound healing’/exp OR ‘wound healing’ OR (wounds: ab, ti AND injuries: ab, ti) OR ‘ulcer*’:ab, ti OR ‘skin ulcer’:ab, ti OR ‘skin lesions’:ab, ti OR ‘tissue repair’:ab, ti OR ‘pressure ulcer’:ab, ti OR ‘diabetic ulcer’:ab, ti OR ‘skin burn’:ab, ti OR ‘wound*’:ab, ti AND #2 ‘light emitting diode*’ OR ‘light-emitting diode’:ab, ti OR ‘led’:ab, ti OR ‘non-coherent light’:ab, ti OR ‘nasa light-emitting diode’:ab, ti OR ‘led therapy’:ab, ti
EMBASE	

### Study selection process

Following the search, article titles and abstracts were initially screened, followed by full-text assessment to determine eligibility. Study selection was conducted independently by two reviewers, and any disagreements were resolved by consensus. When necessary, a third reviewer was consulted to resolve discrepancies, ensuring adherence to the predefined inclusion criteria and the study objectives.

### Data extraction

Data extraction was performed independently by two reviewers, and any discrepancies were resolved through discussion with a third reviewer. The information collected included: title and year of publication, study design, sample size and characteristics (sex and age), type of wound, intervention parameters, application technique, outcome and/or outcome measure(s), follow-up period(s), characteristics of the comparator group, assessed outcomes, and reported results.

### Assessment of methodological quality of randomized trials

The risk of bias of the included studies was independently assessed by two reviewers to ensure the reliability of the process. In cases of disagreement regarding classification, a third reviewer was consulted to reach consensus. For randomized clinical trials, the Cochrane Risk of Bias 2 (RoB 2) tool [23] was used, which evaluates domains related to random sequence generation, allocation concealment, blinding of participants and researchers, blinding of outcome assessment, incomplete outcome data, selective reporting, and other potential sources of bias. Each domain was classified as low risk, some concerns, or high risk of bias, allowing for a detailed assessment of the methodological quality of the studies.

### Data synthesis and analysis

The results were analyzed separately for each outcome of interest. Heterogeneity in the intervention parameters and reported outcomes precluded pooling the outcomes in a quantitative analysis; therefore, the data were summarized through descriptive analysis, presented as percentages, and organized in tables.

### Assessing the credibility of evidence

The Grading of Recommendations, Assessment, Development and Evaluation (GRADE) system [24] was used to assess the quality of evidence, categorizing it into four levels: “high,” “moderate,” “low,” or “very low.” Two investigators independently performed the assessments for each outcome. Randomized clinical trials were initially rated as high certainty by default and were subsequently evaluated for factors that could warrant downgrading the level of evidence based on the following criteria: (1) risk of bias, (2) inconsistency of results, (3) indirectness of evidence, (4) imprecision of estimates, and (5) publication bias.

## Results

**Fig. 1:**
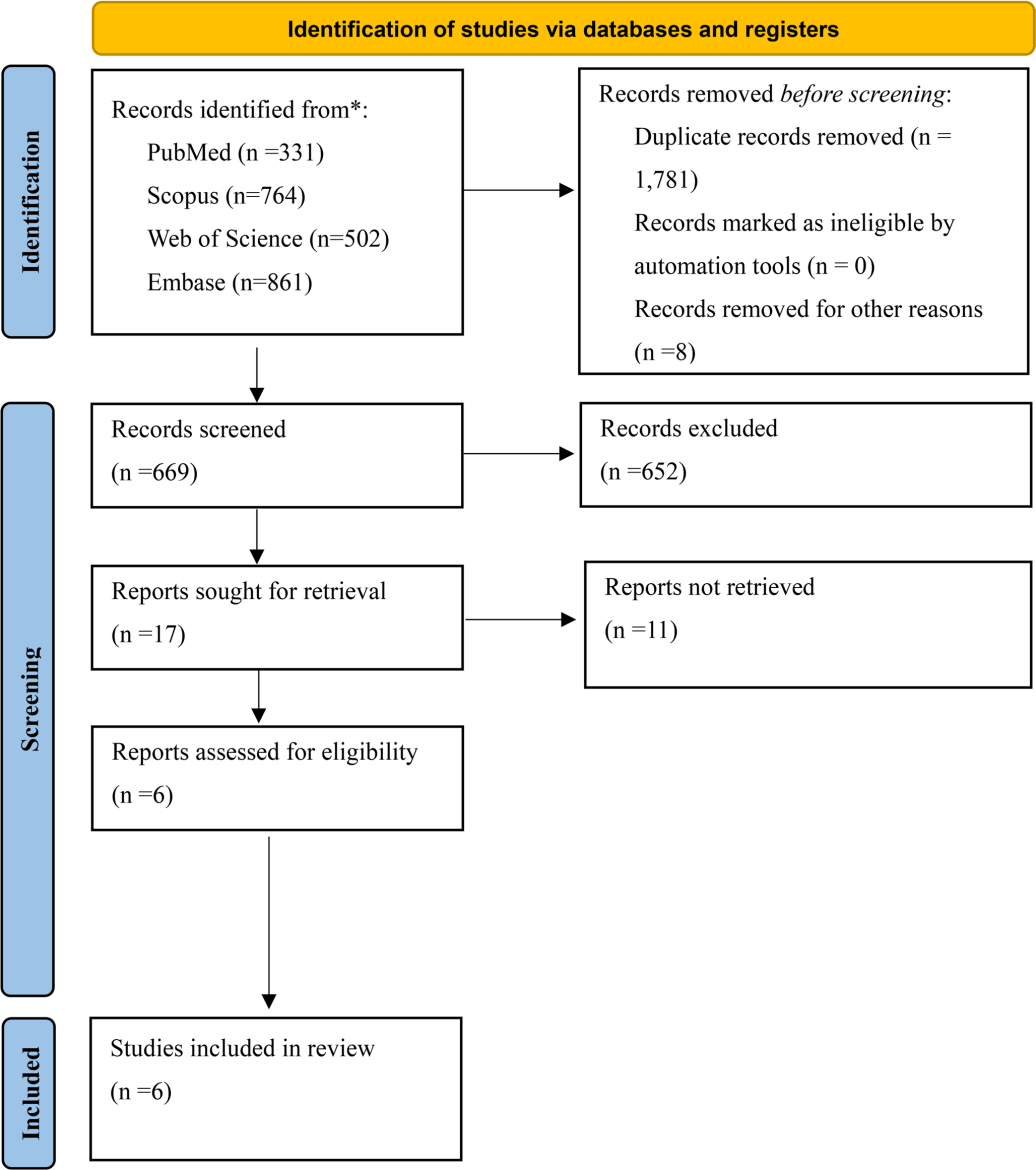
Flowchart of the study selection process according to the Preferred Reporting Items for Systematic Reviews and Meta-Analyses (PRISMA) guidelines

The search identified 2,458 studies. After duplicate removal, 687 records were screened by title and abstract. Of these, 17 reports were sought for retrieval, and six studies met the eligibility criteria and were included in the final analysis. The study selection process is illustrated in Fig. 1.

### Study characteristics

All included studies were randomized clinical trials, although one study was also described as prospective [29]. Sample sizes ranged from 11 to 79 participants, with mean ages between 53.7 and 70.8 years. Most studies investigated diabetic foot ulcers (Grades I–II) [25–27, 29], whereas one study focused on below-knee amputation wounds [28] and another on lower-extremity surgical wounds [30]. Table 3 summarizes the main characteristics and findings of these studies.

**Table 3:** LED phototherapy application parameters used in the included studies

Author/year	Type of wound	Intervention	Control	Characteristics of intervention (wavelength (nm); spot size (cm²); irradiation mode; energy density (J/cm²); output power (mW/W); power density (W/cm² or mW/cm²)	Treatment protocol (irradiation time; application method; frequency and duration of treatment)	Main outcomes
Borges at al, 2024 [25]	Diabetic foot ulcer (Grade I–II)	Red, infrared, and combined LED + standard treatment	Standard treatment + medical follow-up	620–940 nm; spot size: 0.125 cm²; continuous mode; energy density: 6 J/cm²; output power: 0.008–0.009 mW/W	Irradiation time: 73–94 s per point; LED blanket positioned 1 cm from the ulcer and covered with PVC film; perpendicular application; administered daily for 12 weeks (84 sessions) or until complete healing	Red and infrared LEDs did not demonstrate a statistically significant difference; however, both treatments showed a greater magnitude of clinical effect, with a more pronounced trend toward reduction and closure of the lesion area.
Vitoriano et al., 2019 [26]	Diabetic foot ulcer	LED + Standard treatment	LASER	LED 850 nm; spot size: 0.116 cm²; continuous mode; energy density: 5.28 J/cm²; output power: 48 mW; power density: 0.24 W/cm² LASER 830 nm; continuous mode; energy density: 7 J/cm²; output power: 30 mW; power density: 0.25 W/cm²	Irradiation time: 22 s per point; contact application, perpendicular to the tissue; twice weekly for 5 weeks Irradiation time: 28 s per point; contact application, perpendicular to the tissue; twice weekly for 5 weeks	Photobiomodulation with LED and LASER were associated with significant reductions in ulcer size after 10 treatment sessions. Additionally, improvements in neuropathic signs and symptoms were observed with both mod
Frangez et al., 2018 [27]	Diabetic foot ulcer	LED + Standard treatment	Broadband spectrum	625, 660, and 850 nm; pulsed mode; energy density: 2.4 J/cm²	Irradiation time: 5 min per session; non-contact application at a distance of 10 cm from the wound surface; three times weekly for 8 weeks (24 sessions)	Photobiomodulation with LED did not reduce wound area compared with the control group; however, significant improvements in wound bed characteristics were observed between weeks 4 and 8.
Elessawy et al., 2021 [28]	Below-knee amputation	LED + Standard treatment	Standard treatment	650–950 nm; continuous mode; energy density: 4 J/cm²; output power: 50 mW; power density: 50 mW/cm²	Irradiation time: 8 min per session (480 s); direct contact with the wound surface through a transparent film, applied vertically with light pressure; three times weekly for 8 weeks (24 sessions)	Photobiomodulation with LED was associated with greater wound healing and a larger reduction in wound area after eight weeks compared with standard treatment.
Frangez et al., 2017 [29]	Diabetic foot ulcer	LED + Standard treatment	Broadband spectrum	625, 660, and 850 nm; square-wave modulation; energy density: 2.4 J/cm²	Irradiation time: 5 min per session; non-contact irradiation at a distance of 10 cm covering the entire wound area and surrounding tissue; three times weekly for 8 weeks or until complete healing	Photobiomodulation with LED was associated with increased microcirculation and accelerated healing in both diabetic and non-diabetic patients, whereas no improvements were observed in the control groups.
Perper et al., 2020 [30]	Lower-extremity surgical wounds	LED + Standard treatment	Sham+ Standard treatment	633 nm; continuous mode; energy density: 126 J/cm²; power density: 105 mW/cm²	Irradiation time: 20 min per session; non-contact planar array positioned 10 cm from the wound surface; once weekly for 4 weeks	Photobiomodulation with LED did not significantly accelerate healing, with wound closure time remaining similar to that observed in the control group.

*LED* Light-Emitting Diode, *LASER* Light Amplification by Stimulated Emission of Radiation, *CG* Control Group, V: Red LED; IV: Infrared LED; A: Combined LEDs; *GaAlAs* Gallium Aluminum Arsenide; *J/cm²* Joules per square centimeter; cm²: Square centimeter, *mW*: Milliwatt, *W/cm²* Watts per square centimeter, *s* Seconds, *nm* Nanometer, *PVC* Polyvinyl Chloride, *WARP* Wound Healing Acceleration by Radiant Phototherapy (commercial LED device), *NR* not reported

### LED irradiation parameters

The included studies compared the effects of photobiomodulation with LED and low-level laser therapy (LLLT) [26], sham irradiation or standard treatment [25, 30], and only one study used broadband light as a comparator [27, 29]. LED interventions included red emissions (620–633 nm) [25, 30], infrared emissions (850–950 nm) [26, 28], and combined wavelengths (625, 660, and 850 nm) [27, 29]. Energy densities ranged from 2.4 to 126 J/cm², with predominant doses between 4 and 6 J/cm². Output power varied from 0.008 W to 105 mW/cm². Most studies applied LED in continuous mode [25, 26, 28, 30], whereas some used pulsed or square-wave modulation [27, 29]. Irradiation time per session ranged from 22 s to 20 min, and treatment frequency varied from once weekly to daily sessions, with total duration ranging from 4 to 12 weeks (3 to 84 sessions). Application techniques also differed across studies: some applied LED in direct contact with the wound using a transparent film with gentle pressure [28]; others performed non-contact irradiation, maintaining fixed distances of 10 cm from the wound [27, 29, 30]; and one study used a flexible LED pad positioned 1 cm above the ulcer and wrapped in PVC film [25]. A summary of LED application parameters is presented in Table 3.

### Diabetic foot ulcer

#### Wound healing and reduction of wound area

Four studies evaluated wound healing and reduction in the area of diabetic ulcers as outcomes [25–27, 29]. Only one of these did not demonstrate a statistically significant difference compared with the control group; however, a clinically relevant difference was identified, with the LED groups showing a large clinical effect (Cohen’s d > 1.6) compared with the control [25]. In this study, the 940 nm and 620 nm LEDs demonstrated a greater effect on wound area closure when compared with the multispectral 620 + 940 nm LED [25]. Two other studies [27, 29] investigated multispectral devices combining red and infrared wavelengths and reported reductions in wound area after four and eight weeks of treatment [27, 29]. In a comparative study between infrared LED and infrared LASER, both devices promoted a healing effect. However, the laser proved to be more effective, achieving a healing rate of 81.17%, whereas the LED reached 62.26% [26].

#### Wound bed quality

Two studies assessed wound bed quality using the Falanga score [27, 29]. Wound bed quality remained similar at baseline and after two weeks in both the multispectral LED group (625, 660, and 850 nm; 2.4 J/cm²) and the control group. However, beginning in the fourth week, LED therapy promoted a significant improvement in wound bed quality, an effect that was sustained and intensified through the eighth week, as evidenced by increased granulation tissue formation and reduced fibrin and eschar compared with the control group.

#### Microcirculation

One study assessed microcirculation using LASER Doppler flowmetry (LD flux) and thermophototherapy [29]. After eight weeks, multispectral photobiomodulation with LED (625, 660, and 850 nm; 2.4 J/cm²) promoted a significant increase in blood flow around the wounds of diabetic (+ 0.42) and nondiabetic (+ 0.64) patients compared with the control group (+ 0.24).

#### Temperature

One study assessed skin temperature using thermography [25]. Irradiation with a 940 nm infrared LED (6 J/cm²) and a 620 nm red LED (6 J/cm²) did not produce changes in skin temperature, either throughout the treatment period or at the 30-day follow-up [25].

#### Neuropathic symptoms

Photobiomodulation with LED appears to be able to modulate perilesional sensitivity and improve the signs and symptoms of peripheral neuropathy associated with diabetic wounds [25, 26]. One study assessed perilesional sensitivity using a digital esthesiometer [25], whereas another evaluated signs and symptoms of peripheral neuropathy through neuropathic symptom and sign scores [26]. Irradiation with infrared LED (940 nm, 6 J/cm²) increased perilesional pain compared with the control group, whereas multispectral LED (620 nm + 940 nm, 6 J/cm²) reduced pain within 30 days after the intervention [25]. Neuropathic signs (Achilles reflex, vibratory sensitivity, pain sensitivity, and thermal sensitivity), as well as patient-reported symptoms—including burning, tingling, numbness, cramps, and pain—showed improvement following treatment with 850 nm LED (5.28 J/cm²) and 830 nm laser (7 J/cm²) compared with conventional therapy [26].

#### Adverse effects

The four studies that reported the use of photobiomodulation with LED in lesions of individuals with diabetes mellitus did not report adverse effects in participants during treatment or up to 30 days after completion of the intervention [25, 27, 29, 30].

### Lower-extremity surgical wounds

#### Wound healing and reduction of wound area

Two studies evaluated complete healing and reduction in wound area in individuals with lower-extremity surgical wounds [28, 30]. For these lesions, high-dose red LED at 633 nm (126 J/cm²) did not demonstrate benefit at the end of four weeks, showing only transient improvement between the second and third weeks and remaining comparable to conventional treatment [30]. In contrast, multispectral LED at 650–950 nm (4 J/cm²) promoted greater reduction in wound area and higher healing rates over eight weeks compared with conventional treatment [28].

#### Healing time

One study followed participants until complete healing of lower-extremity surgical wounds [30]. High-dose red LED at 633 nm (126 J/cm²) did not reduce re-epithelialization time, as the treated group showed a mean healing time of 63.2 ± 12.2 days, whereas the control group healed in 48.7 ± 11.1 days [30]. These findings suggest that, under the evaluated conditions, high-dose 633 nm red LED does not promote accelerated healing and may be associated with a longer re-epithelialization time. The evidence-informed parameters for photobiomodulation with LED in chronic lower-limb wounds are summarized in Table 4.

**Table 4:** Evidence-informed parameters for LED therapy in chronic lower-limb wounds

Parameter	Evidence-Based Range	Evidence Overview
Energy density (J/cm^2^)	2.4–10 J/cm²	Most clinical benefits were observed within this dose range in both DFUs and surgical wounds; doses ≥ 100 J/cm² showed no benefit or were associated with delayed healing
Wavelength (nm)	Red (620–660 nm) and Near-infrared (850–950 nm)	Both spectra demonstrated clinical effects on wound healing, microcirculation, and peripheral neuropathic symptoms
Spectral mode	Single or multispectral	Multispectral LEDs demonstrated benefits when appropriate dosimetry was applied, but no consistent superiority over single-wavelength LEDs was observed
Power density (W/cm²)	≤ 100 mW/cm²	Lower irradiances were associated with favorable biological responses without inducing thermal effects
Application mode	Contact or non-contact (≤ 10 cm)	Effective outcomes were reported with both techniques when energy delivery was properly controlled
Session duration	30 s to 8 min per point	There was wide variability, with efficacy linked to dose rather than exposure time alone
Treatment frequency	2–3 sessions per week	The most effective protocols applied LED therapy at least twice weekly
Total treatment period	≥ 4 weeks	Improvements in wound area, wound bed quality, and microcirculation were typically observed after 4–8 weeks of treatment

*DFUs* diabetic foot ulcers, *J/cm²* joules per square centimeter, *nm* nanometer; *NIR* near-infrared, *W/cm²* watts per square centimeter, *mW/cm²* milliwatts per square centimeter

#### Results of the methodological quality assessment

**Fig. 2:**
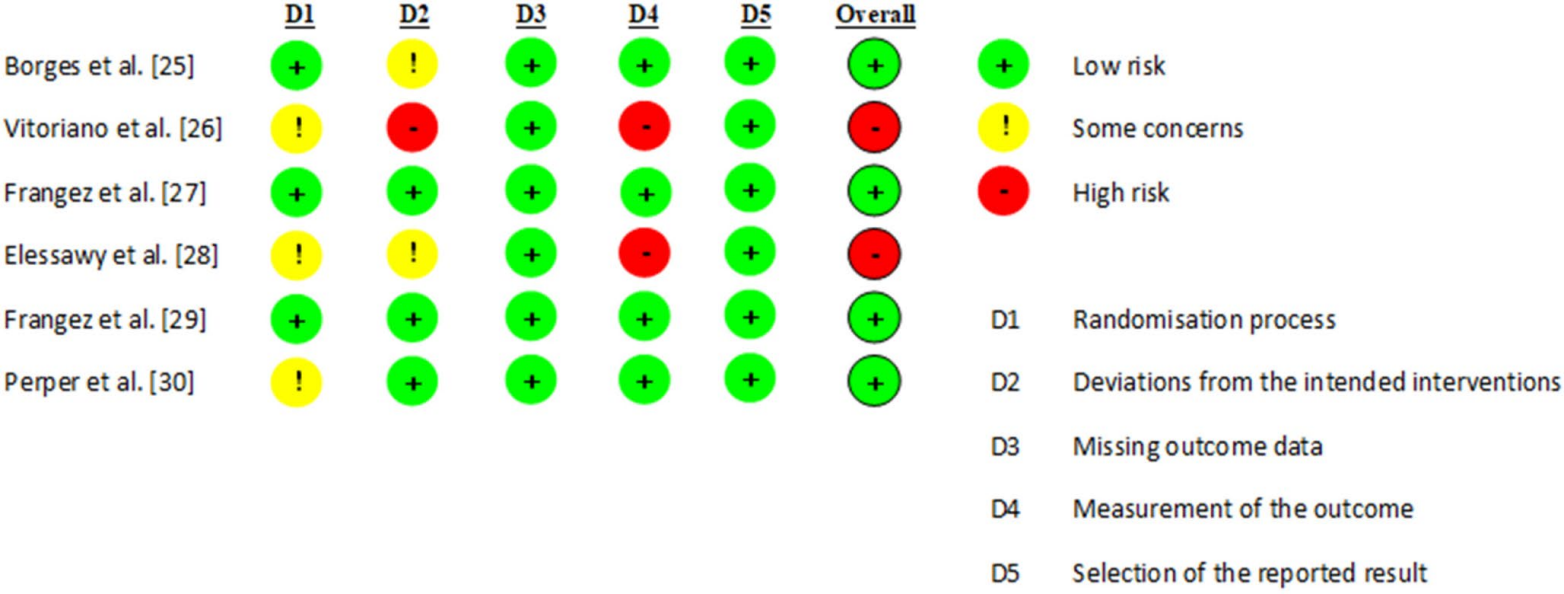
Assessment of the risk of bias of the included studies according to the Risk of Bias 2 (RoB 2) tool

**Fig. 3:**
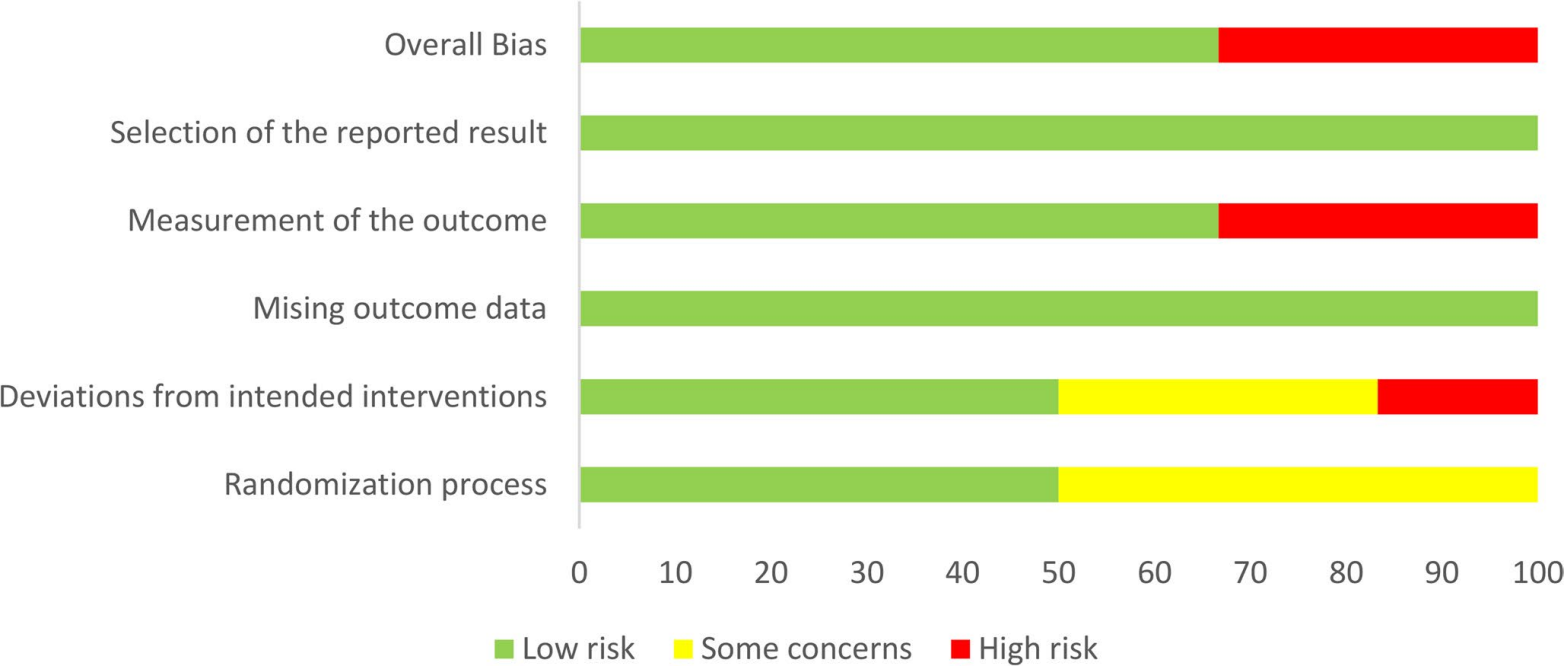
Bar plots of risk of bias for included studies

The methodological quality of the included studies was assessed using the Risk of Bias 2 (RoB 2) tool, as presented in Figs. 2 and 3. Four studies were classified as having a low risk of bias, while two studies were classified as high risk. The methodological quality of the included studies was assessed using the Risk of Bias 2 (RoB 2) tool, as shown in Figs. 2 and 3. Overall, four studies were classified as having a low risk of bias, while two studies were classified as having a high risk of bias. A qualitative synthesis indicated that the concerns were mainly related to the randomization process (D1), deviations from planned interventions (D2), and risk of bias in outcome measurement (D4).

#### Results of the certainty of the evidence

The overall certainty of evidence for comparisons between LED photobiomodulation and control groups was rated as very low for both outcomes assessed. For diabetic foot ulcer healing, the certainty of evidence was downgraded due to very serious inconsistency, serious indirectness, and serious imprecision. For surgical wound healing in the lower limbs, the certainty of evidence was downgraded due to a serious risk of bias, very serious inconsistency, serious indirectness, and serious imprecision. GRADE ratings of overall certainty of evidence are presented in Table 5.

**Table 5:** GRADE – evaluation of evidence

Certainty assessment							№ of patients		Effect		Certainty	Importance
№ of studies	Study design	Risk of bias	Inconsistency	Indirectness	Imprecision	Other considerations	LED	standard care or LASER	Relative (95% CI)	Absolute (95% CI)		
Healing of diabetic foot ulcers (follow-up: mean 8 weeks)												
4	randomised trials	not serious	very serious^a^	serious^b^	serious^c^	none	91	69	-	see comment	⊕◯◯◯ Very low^a, b,c^	IMPORTANT
Healing of Lower-extremity surgical wounds (follow-up: mean 6 weeks)												
2	randomised trials	serious^d^	very serious^a^	serious^b^	serious^c^	none	22	22	-	see comment	⊕◯◯◯ Very low^a, b,c, d^	IMPORTANT

*CI* confidence interval; (a) Variability in the direction of effects was observed among the included studies, justifying a downgrade in the certainty of the evidence; (b) The research question is not directly addressed by the included studies due to differences in the interventions, justifying a downgrade in the certainty of the evidence; (c) The certainty of the evidence was downgraded by one level due to imprecision related to the small sample size; (d) The certainty of evidence was downgraded by one level due to the high risk of bias identified in one of the studies included in this analysis

## Discussion

**Fig. 4:**
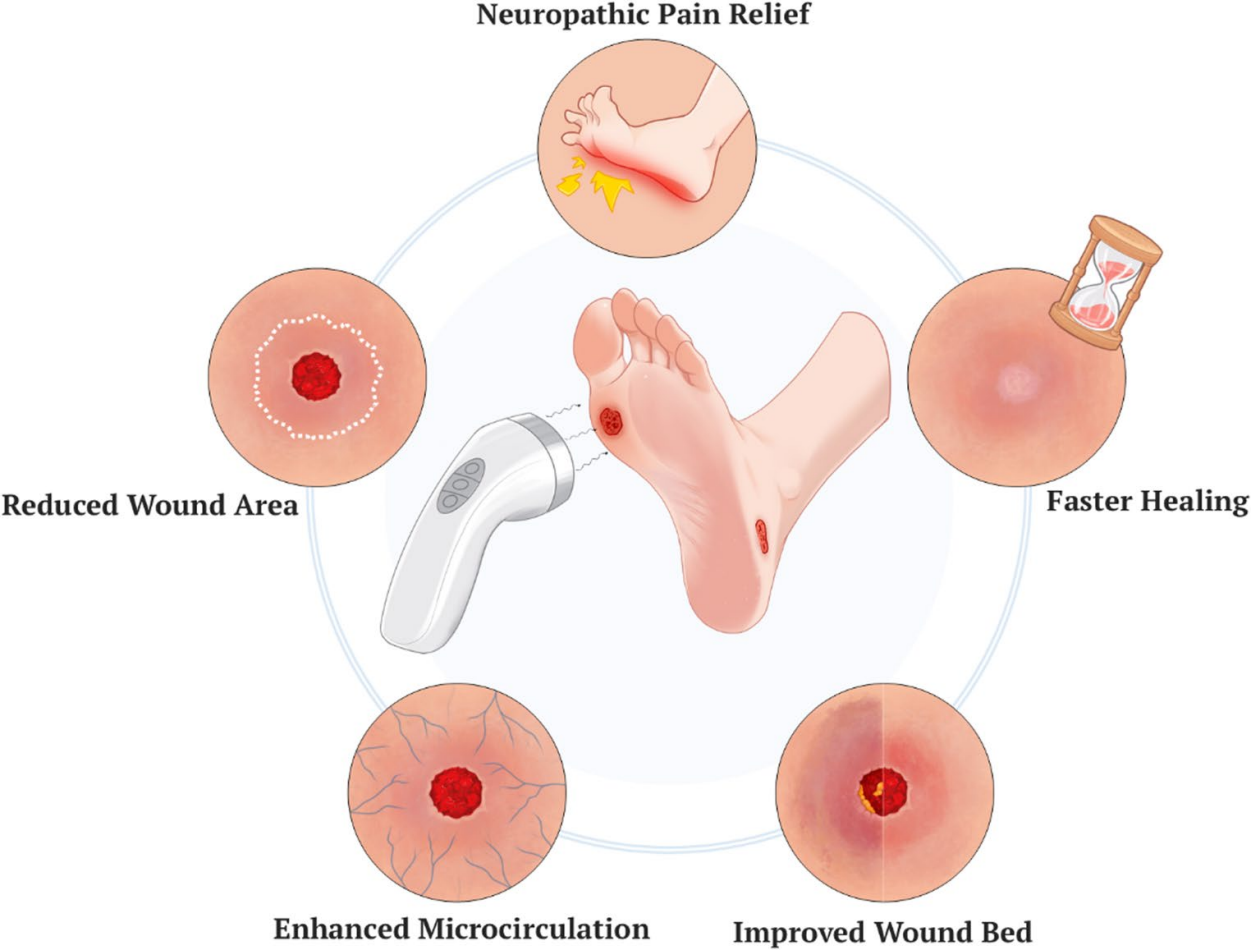
Beneficial effects of led therapy on wound healing

This review aimed to analyze the effects of LED photobiomodulation on cutaneous wound healing. Overall, the included studies suggest that LED photobiomodulation may exert beneficial effects on the wound healing process, although the certainty of the evidence was classified as very low. The included studies reported a potential reduction in wound area, shorter healing time, and improvements in wound bed characteristics. In addition, attenuation of neuropathic symptoms and increased local microcirculation were observed, particularly in individuals with diabetic foot ulcers. These positive effects appeared to be predominantly associated with low energy densities (Fig. 4).

Due to the absence of essential statistical data in several included studies, it was not possible to perform a quantitative synthesis of the findings. Missing information included means, standard deviations, measures of variability, confidence intervals, and complete numerical outcome data. In addition, many studies reported outcomes only descriptively or graphically, preventing accurate effect size estimation and data pooling. Therefore, a qualitative synthesis was conducted to ensure a methodologically appropriate interpretation of the available evidence.

The healing effects observed in clinical studies using LED photobiomodulation may be mediated by the activation of molecular pathways triggered by photon absorption in target tissues [31, 32]. These pathways are associated with angiogenesis, cellular proliferation, collagen synthesis, and cell migration [31, 32]. However, these effects depend on how light energy is absorbed by the tissue and are modulated by device parameters, including wavelength (nm), energy dose (J/cm²), irradiance (mW/cm²), and application time, as well as by the optical properties of the target tissue [33, 34].

Among these parameters, energy dose refers to the total light energy delivered per unit area (J/cm²), determined by variables such as device power, exposure time, and irradiated area [35]. This parameter is particularly relevant because it influences the amount of energy effectively delivered to the target tissue, directly affecting biological and therapeutic responses [36, 37]. Therefore, inadequate parameter adjustment may lead to variable therapeutic outcomes, even when similar wavelengths are used.

**Fig. 5:**
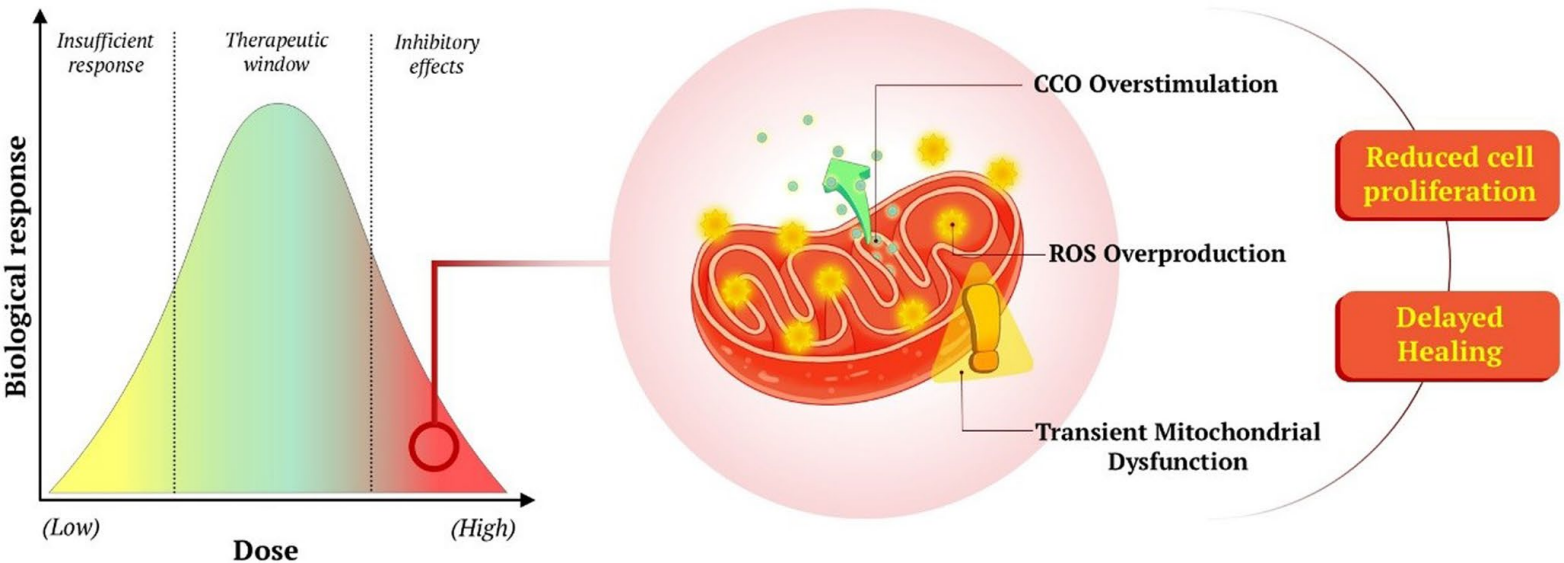
Therapeutic window and dose-dependent biological effects of photobiomodulation

The present review observed that the only study reporting unfavorable clinical outcomes used an energy density of 126 J/cm² [30]. This finding may be related to the high dose applied, suggesting a dose-dependent response in accordance with the Arndt–Schulz law, which supports the concept of a therapeutic window. Thus, irradiation above the proposed therapeutic window may exert inhibitory effects, potentially related to overstimulation of cytochrome c oxidase (CCO), increased production of reactive oxygen species (ROS), oxidative stress, and transient mitochondrial dysfunction [38]. These events may ultimately result in reduced cellular proliferation and delayed tissue repair, indicating that higher doses do not necessarily translate into greater clinical efficacy [38] (Fig. 5).

Biphasic dose–response model of photobiomodulation. Within the therapeutic window, moderate mitochondrial stimulation promotes adenosine triphosphate (ATP) synthesis and tissue repair. At supratherapeutic doses, excessive mitochondrial stimulation may increase ROS production, promote transient redox imbalance, and impair cell proliferation and wound healing. Adapted from previous studies [38, 39].

The concept of a therapeutic window may also explain the absence of differences among the wavelengths used. These similar results do not indicate that wavelength selection is irrelevant; rather, they may suggest that the selected spectra were within the therapeutic window capable of eliciting a clinical response. Preclinical studies indicate that red light spectra (≈ 620–660 nm) and near-infrared spectra (≈ 800–900 nm) exhibit partial overlap with the absorption peaks of CCO, which is considered the primary mitochondrial chromophore involved in mediating the effects of PBM [39].

In addition to its healing effects, studies suggest that LED photobiomodulation may modulate neuropathic symptoms [25–29]. This effect may be associated with pathophysiological mechanisms underlying diabetic peripheral neuropathy (DPN), such as inflammation and reduced axonal blood flow, which may be modulated by PBM [40, 41]. Recent systematic reviews have demonstrated that PBM may improve pain and nerve conduction in individuals with DPN [42, 43].

DPN and microvascular dysfunction are key factors in the development, recurrence, and chronicity of ulcers [44]. Clinically, interventions that improve protective sensation and tissue perfusion may serve as adjunctive therapies by supporting a more favorable environment for wound healing [45]. However, because the studies included in this review assessed neuropathic outcomes in isolation, their relationship with recurrence or long-term complications remains unclear.

Chronic wounds represent a major clinical challenge, characterized by delayed healing, an increased risk of infection, and significant impacts on individual functionality and healthcare-related costs [46]. Although this systematic review suggests beneficial effects of LED photobiomodulation, important uncertainties remain regarding the establishment of optimal treatment parameters. The certainty of the evidence was rated as very low due to very serious inconsistency, indirectness, and imprecision. For lower-limb surgical wounds, the certainty was further downgraded due to serious risk of bias. Therefore, these findings should be interpreted with caution in light of the methodological limitations and low certainty of the available evidence.

In addition, the included studies were characterized by small sample sizes, heterogeneity in irradiation protocols and outcome measures, and methodological limitations identified in the risk-of-bias assessment. These limitations included concerns related to the randomization process, deviations from intended interventions, and outcome assessment, further reducing confidence in the reproducibility and generalizability of the findings.

To strengthen the evidence base for this therapeutic approach, future randomized clinical trials should prioritize greater methodological standardization, including clear and reproducible descriptions of irradiation parameters, in order to enhance the reliability of findings. In addition, dose–response study designs, stratification according to lesion type and severity, and the adoption of standardized clinical and functional outcomes are recommended to improve the comparability and reproducibility of results. These outcomes may include percentage reduction in wound area, time to complete healing, and measures of microcirculation, neural regeneration, and quality of life.

### Limitations and strengths of the study

This study presents several important limitations that should be considered when interpreting the results. The included studies demonstrated methodological problems in areas of risk of bias assessment, including issues related to the randomization process, deviations from planned interventions, and outcome assessment. Consequently, the available evidence should be interpreted with caution. Furthermore, the small number of included studies and the limited sample sizes restrict the robustness and generalizability of the conclusions. The lack of sufficient statistical information prevented data pooling and quantitative meta-analysis, limiting this review to a qualitative synthesis. Moreover, energy density was the parameter that showed the greatest variability among the trials, ranging from 2.4 to 126 J/cm², while the other parameters showed relatively narrower ranges. This variability makes it difficult to identify an ideal therapeutic window and reinforces the need for greater methodological consistency among the studies. Although definitive standardization of protocols is not yet possible based on the currently available evidence, the results of this review may provide clinically relevant guidance and help establish directions for future research focused on optimizing and standardizing therapeutic protocols.

## Conclusion

The available evidence suggests that LED photobiomodulation may promote tissue repair in lower-limb surgical wounds and in patients with diabetes, potentially contributing to faster wound healing and structural improvement of the wound bed. In addition, the intervention may be associated with improved microcirculation and relief of neuropathic symptoms in individuals with diabetic foot ulcers.

## Data Availability

The data that support the findings of this study are available from the corresponding author upon reasonable request.

## Declarations

### Permission to reproduce material from other sources

This manuscript does not contain any previously published material from other sources.
